# Estimation of ionic currents and compensation mechanisms from recursive piecewise assimilation of electrophysiological data

**DOI:** 10.3389/fncom.2025.1458878

**Published:** 2025-03-04

**Authors:** Stephen A. Wells, Paul G. Morris, Joseph D. Taylor, Alain Nogaret

**Affiliations:** Department of Physics, University of Bath, Bath, United Kingdom

**Keywords:** parameter estimation, dynamical systems, data assimilation, ion channels, neurons and networks

## Abstract

The identification of ion channels expressed in neuronal function and neuronal dynamics is critical to understanding neurological disease. This program calls for advanced parameter estimation methods that infer ion channel properties from the electrical oscillations they induce across the cell membrane. Characterization of the expressed ion channels would allow detecting channelopathies and help devise more effective therapies for neurological and cardiac disease. Here, we describe Recursive Piecewise Data Assimilation (RPDA), as a computational method that successfully deconvolutes the ionic current waveforms of a hippocampal neuron from the assimilation of current-clamp recordings. The strength of this approach is to simultaneously estimate all ionic currents in the cell from a small but high-quality dataset. RPDA allows us to quantify collateral alterations in non-targeted ion channels that demonstrate the potential of the method as a drug toxicity counter-screen. The method is validated by estimating the selectivity and potency of known ion channel inhibitors in agreement with the standard pharmacological assay of inhibitor potency (IC50).

## 1 Introduction

Parameter estimation methods build models of biological neurons and circuits from experimental time series data (O’Leary et al., 2015; Van Geit et al., 2008). Different computational approaches have been used ranging from linear-regression (Prinz et al., 2003; Golowasch et al., 2002; Ahrens et al., 2005), to evolutionary algorithms (Achard and De Schutter, 2006; Buhry et al., 2012; Lynch and Houghton, 2015), genetic algorithms (Brookings et al., 2014; Nandi et al., 2022), simulated annealing (Ye et al., 2015), path integrals (Kostuk et al., 2012), Kitagawa state space modelling (Vavoulis et al., 2012; Kitagawa, 1998), and Lagrangian optimization of Hodgkin-Huxley models (Wächter and Biegler, 2006; Nogaret et al., 2016; Meliza et al., 2014; Armstrong, 2020). The latter approach synchronizes the mathematical equations of the model to time series data to obtain the optimal set of parameters. Once the training period is complete, the mathematical equations configured with the optimal parameters will predict the dynamics of state variables. Some state variables are observed, such as the membrane voltage, which can then be compared to measured time series. This provides an important validation point of the completed model. Other state variables, such as the gating variables of the Hodgkin-Huxley model, or the ionic currents, are inaccessible to time series measurements, hence the difficulty of fully validating model predictions. The estimated parameters include the activation thresholds, ionic conductances, and gate recovery time constants which can identify sub-types of different classes of ion channels and help classify different neuron types (Chan et al., 2011; Migliore and Shepherd 2005; Molyneaux et al., 2007; Zeng and Sanes 2017). Therefore, if a parameter estimation method were validated on biological data, it could then be used to determine the full complement of ion channels of a neuron including their response to drugs. In practice, the lack of knowledge about the true equations of biological neurons is one factor limiting our confidence in the parameter solutions.

Here, we discuss two recent studies that introduce the RPDA method and validate it experimentally in pharmacologically altered neurons. The RPDA method was introduced by Wells et al. (2024) to reliably predict ionic current waveforms in the presence model error. This approach is called for by the empirical nature of Hodgkin-Huxley models and by the need to mitigate the effect of model error on the estimated quantities. The predictive power of the RPDA method was experimentally tested in a separate study by Morris et al. (2024). They used RPDA to evaluate the selectivity and potency of known ion channel antagonists applied to block specific ion channels in hippocampal neurons (CA1). The RPDA method was able to predict the change in ionic charge flowing across the ion channel targeted by the antagonist, in agreement with IC50 calibration of drug potency. Because RPDA evaluates changes in all ion channels in one go, it was also able to quantify compensation effects in ion channels not targeted by the antagonist. This demonstrates the potential of RPDA as a high throughput drug toxicity counter-screen. One aim of this paper is thus to present the RPDA method and its experimental validation in one narrative linking the key findings of the detailed computational and pharmacological studies. This is important to show that parameter estimation can infer reliable information from biological systems and not only surrogate data. This paper also allows us to discuss the criteria that current protocols need to fulfill to extract a maximum of information from biological neurons.

## 2 Experimental protocol

For all model parameters to be constrained by the neuron output, it is necessary that the current stimulation protocol is sufficiently informative (Wells et al., 2024). Unlike voltage clamps whose stimulation protocols have been optimized (Groenendaal et al., 2015; Lei et al., 2023; Beattie et al., 2018) less effort has been directed to optimizing current protocols. The current stimulation must be designed to elicit a response in the depolarised, sub-threshold and hyperpolarized states of the neuron to constrain the parameters that determine each of these oscillation modes. It will include positive depolarising current pulses, small amplitude current oscillations causing the membrane voltage to oscillate about the resting potential, and negative current pulses. The current dynamics will incorporate a range of fast and slow-varying oscillations covering the internal time constants of the neuron: [0.1 ms, 500 ms]. If this criterion is only partially met, parameter estimation will produce multivalued solutions. For example, a tonic current would fail to elicit enough information from the neuron to constrain all 67 model parameters, in addition to causing membrane voltage oscillations lacking in reproducibility (Mainen and Sejnowski, 1995). A good current protocol is one that elicits different responses to different parameter values.

**Figure 1:**
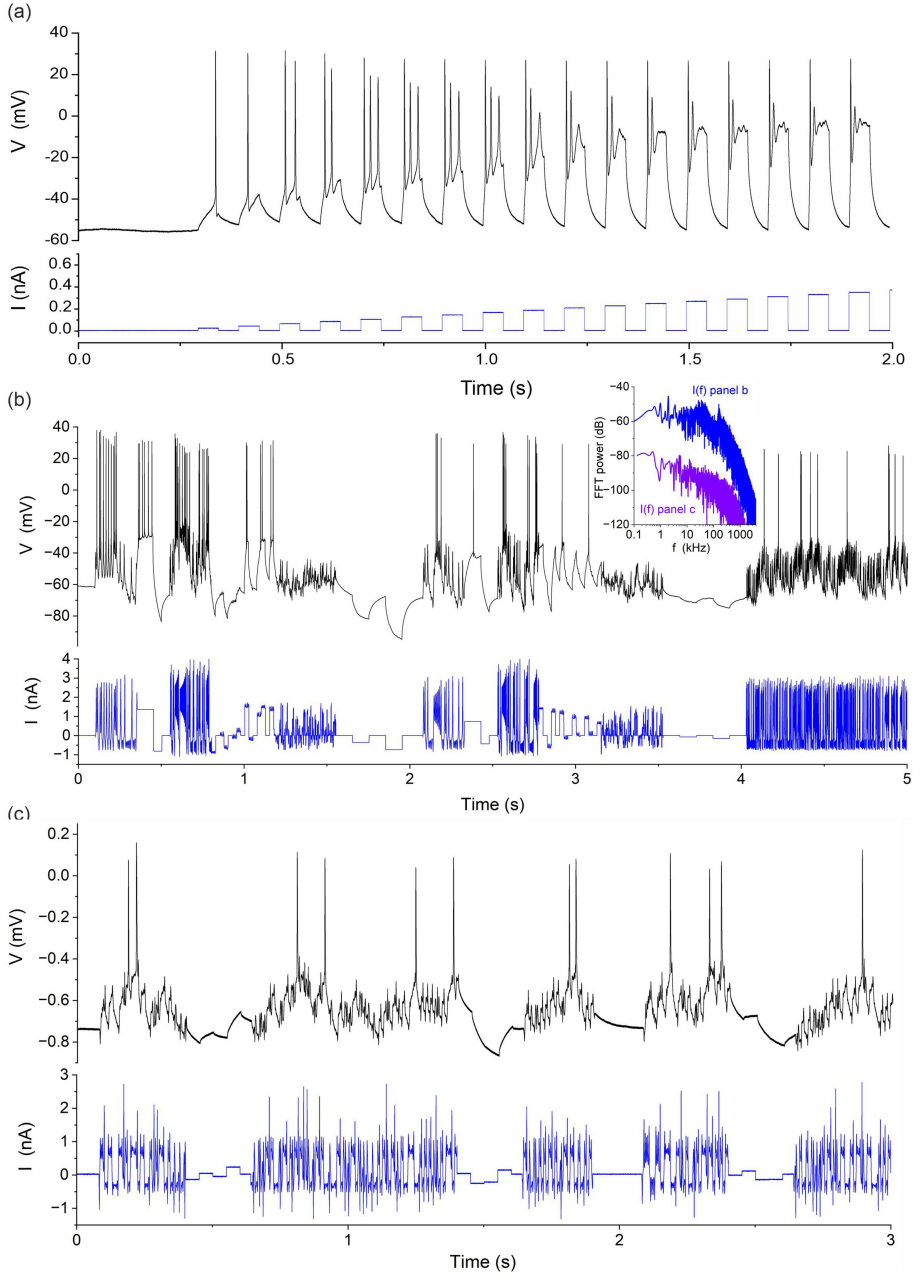
Stimulation protocols applied to rodent hippocampal neurons (CA1). **(A)** Calibration protocol consisting of a sequence of current steps of increasing amplitude. **(B)** Protocol mixing a chaotic signal (Lorenz) and random current steps. **(C)** Protocol mixing a hyperchaotic current and random steps. *Inset:* Power spectra *I*(*f*) of current protocols in panels **(B)** and **(C)**.

We optimised the stimulation protocol in the two steps shown in Figures 1A–C. We first applied a calibration protocol consisting of square steps of increasing amplitude to determine the optimal gain setting of the current clamp amplifier (Figure 1A). For the CA1 hippocampal neuron measured here, the optimal stimulation range is 0.1 to 0.25 nA. Currents outside this range elicit too few action potentials, due to insufficient stimulation when *I* < 0.1 nA and due to depolarization block when *I* > 0.25 nA. We then constructed current protocols that meet the informative criteria described above, and that apply intermediate currents eliciting the maximum number of action potentials per assimilation window. Figure 1B shows a current protocol that combines square pulses with chaotic oscillations produced by the Lorenz system:


(1) $$\left\{ \begin{array}{l} x=\sigma \left(y-z\right),\\ y=x\left(\rho -z\right)-y,\\ z=xy-\beta z\text{.} \end{array}\right.$$

with $\sigma =10,\beta =\frac{8}{3},\rho =28.$ Initial conditions of Equation 1 were: x=y=z=1. Figure 1C shows a hyperchaotic current protocol mixed with current steps. This used the following map with two positive Lyapunov exponents to generate the current oscillations:


(2) $$\left\{ \begin{array}{l} x=x\left(1-y\right)+\alpha z,\\ y=\beta \left(x^{2}-1\right)y,\\ z=\gamma \left(1-y\right)t,\\ t=\eta z\text{.} \end{array}\right.$$

with α=−2,β=1,γ=0.2,η=1. Initial conditions of Equation 2 were: x=y=z=t=1. Both systems are equally suitable since they elicit multiple action potentials while probing neuron dynamics in the subthreshold regime near the resting potential and the hyperpolarized regime with negative current pulses. Fourier spectra of the current protocols in Figures 1B,C (inset to Figure 1) show a low pass response with cut-off frequencies of 50 kHz and 200 kHz, respectively. These protocols effectively probe the [0.01 ms, 500 ms] range of gate recovery times across the complement of ion channels of most neurons. We also know that the stimulus in Figure 1B is sufficient to constrain all parameters of our neuron model because we used it to successfully estimate back the correct model parameters from time series data generated by this model. The worst deviation of a parameter estimate from true value was 0.65%. Most parameters were recovered to within less than 0.01% error (Wells et al., 2024). This proves that the current protocol in Figure 1B satisfies the identifiability criterion and that, here, the uncertainty on parameter estimates will mainly arise from model error and the lack of knowledge about the true biological model.

**Figure 2:**
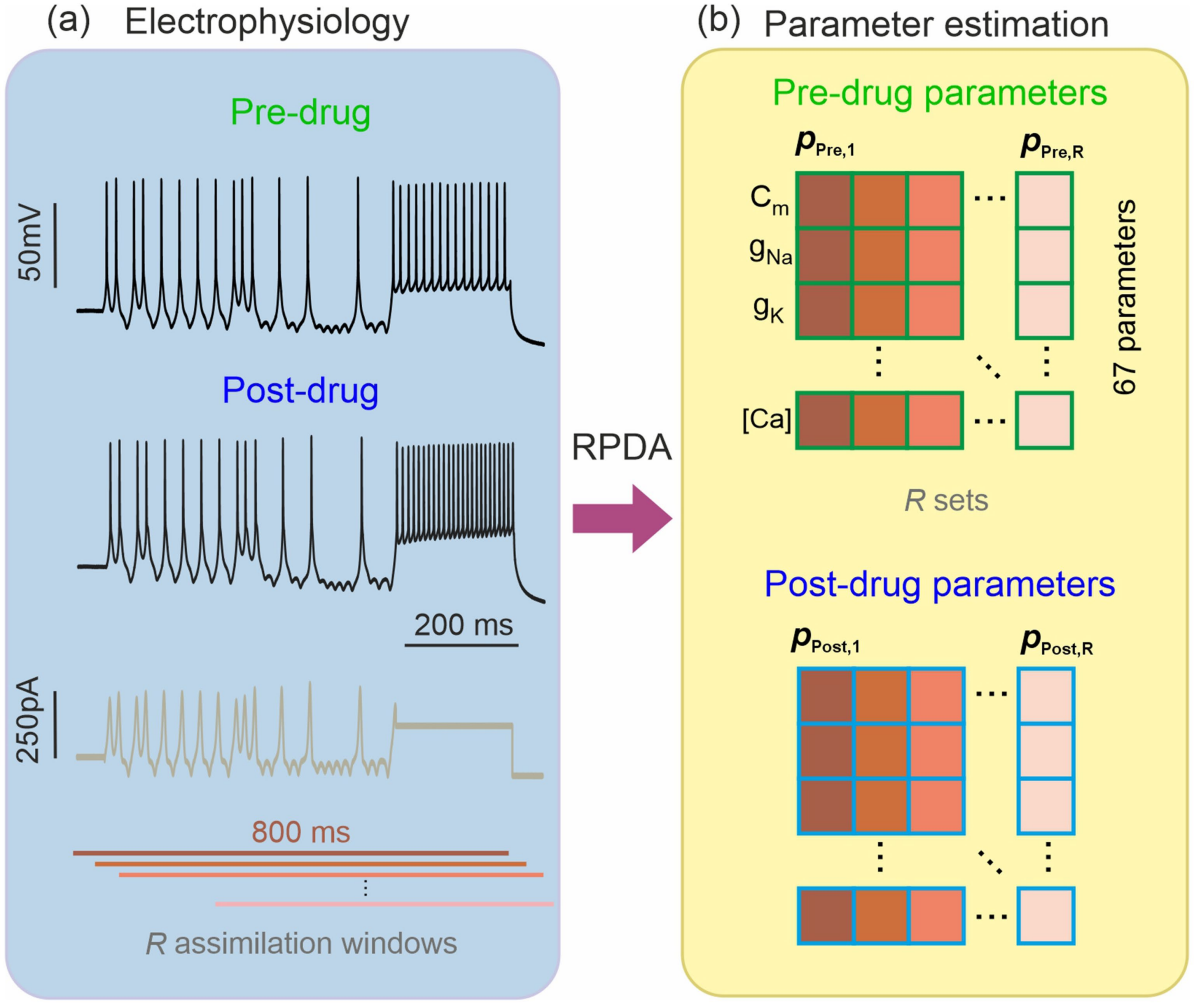
Estimation of the ion channel parameters in a pharmacologically altered neuron. **(A)** Hippocampal neuron recorded before and after blocking an ion channel with an antagonist of known selectivity and molar concentration (potency). The pre-drug and post-drug recordings are stimulated by the same current protocol (brown trace). **(B)** Recursive Piecewise Data Assimilation (RPDA) estimates 67 parameters by synchronizing the neuron-based conductance model to an 800 ms long recording of the membrane voltage. Rather than a single set, we constructed a statistical sample of *R* parameter sets by assimilating *R* 800 ms long windows offset from by 20 ms. We thus obtained *R*-parameter sets from pre-drug data {$p_{1,\ldots ,R}^{Pre}$} and *R*-parameter sets from post-drug data {$p_{1,\ldots ,R}^{\mathrm{Post}}$}.

We performed whole-cell recordings of CA1 neurons in acute brain slices from Han Wistar rats at P15-17. Following decapitation, the brain was removed and placed into an ice-cold slicing solution composed of (mM): NaCl 52.5; sucrose 100; glucose 25; NaHCO_3_ 25; KCl 2.5; CaCl_2_ 1; MgSO_4_ 5; NaH_2_PO_4_ 1.25; kynurenic acid 0.1, and carbogenated using 95% O_2_/5% CO_2_. A Campden 7,000 smz tissue slicer (Campden Instruments UK) was used to prepare transverse hippocampal slices at 350 μm, which were then transferred to a submersion chamber containing carbogenated artificial cerebrospinal fluid (aCSF) composed of (mM): NaCl 124; glucose 30; NaHCO_3_ 25; KCl 3; CaCl_2_ 2; MgSO_4_ 1; NaH_2_PO_4_ 0.4 and incubated at 30°C for 1–5 h prior to use. Synaptic transmission was inhibited pharmacologically in order to remove random synaptic inputs from the surrounding network. To this end all experiments were performed in the presence of (μM) kynurenate 3, picrotoxin 0.05, and strychnine 0.01, to inhibit ionotropic glutamatergic, *γ*-aminobutyric acid (GABA)-ergic, and glycinergic neurotransmission, respectively. The slices were transferred on the stage of an Axioskop 2 upright microscope (Carl Zeiss) to identify pyramidal CA1 neurons from their morphology and location using interference contrast optics. The chamber was perfused with a carbogenated solution aCSF (as above) at 2 mL.min^−1^ at 30 ± 1°C. Patch pipettes were pulled from standard walled borosilicate glass (GC150F, Warner Instruments) to a resistance of 2.5–4 MΩ and filled with an intracellular solution composed of (mM): potassium gluconate 130; sodium gluconate 5, HEPES 10; CaCl_2_ 1.5; sodium phosphocreatine 4; Mg-ATP 4; Na-GTP 0.3; pH 7.3; filtered at 0.2 μm. CA1 neurons were recorded with a current clamp amplifier (Molecular devices, MultiClamp 700B) driven by a Labview controller (National Instruments) via a 16bit USB-6363 DAQ card (National Instruments). The Labview controller delivered the current protocol injected in the neuron (Figure 1) and recorded the neuron membrane voltage. The sampling rate was 100 kHz.

## 3 Recursive piecewise data assimilation

The Recursive Piecewise Data Assimilation (RPDA) Algorithm we now describe resolves several convergence issues in data assimilation (Wächter and Biegler, 2006). One issue compromising convergence is the multiplicity of local minima in the cost function to minimize. Convergence to these false solutions reduces the success rate of conventional data assimilation. This is increasingly problematic when optimizing large problems involving multiple (>12) ion channels. Either the parameter search fails or the solution become multi-valued upon the choice of different starting points. These issues are exacerbated when the model equations are unknown, which is unavoidable when modelling biological neurons. The empirical Hodgkin-Huxley models are one such model. Their approximate nature is responsible for turning local/global minima into long deep valleys in the cost function landscape along which parameter correlations develop. The principle of RPDA is to reinject data into the constraints of Lagrangian optimization to bias convergence towards the true solution (Wells et al., 2024; Zimmer and Sahle, 2015). RPDA assimilates blocks of data in a piecewise manner across the assimilation window. It reinjects membrane voltage data in the constraints at the beginning of each block of data locally relaxing the constraints to apply a bias towards the solution. This bias is progressively released as the block size is increased from one iteration to the next while the parameter solution at the previous iteration is used as the starting point to the next, hence keeping the memory of the bias. When the block size becomes equal or greater than the size of the assimilation window, the optimal solution is obtained. If the initial block size is too small, the bias to convergence may cause convergence to fail. In which case the starting block size is incremented and the recursive data assimilation restarts from the larger block size. A second advantage of RPDA is that the reinjected data perturb the fitting landscape. This perturbation impedes the formation of minima in the fitting landscape allowing the solution to remain a good solution and to be improved with each iteration. The idea here is similar to noise regularization which has been shown to improve convergence towards the global minimum. The successful convergence of RPDA was validated in different scenarios using different initial conditions, or different current protocols. In all cases, RPDA recovered the true parameters of the typical neuron-based conductance model. For well-posed problems, RPDA was found to converge 100% of the time as shown by Wells et al. (2024).

**Algorithm:**
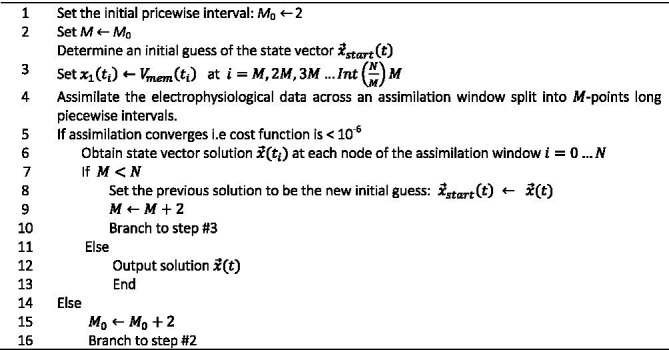
Recursive piecewise data assimilation.

RPDA minimizes a least-square cost function which measures the misfit between a state variable representing the membrane voltage, $x_{1}$, and the experimental membrane voltage, $V_{mem}$, recorded at discrete times *t*_i_, $i\in \left[0,N\right]$ spanning the assimilation window of duration *T*:


(3) $$c\left(\vec{x}\left(0\right)\right)=\frac{1}{2}\;\overset{N}{\underset{i=0}{\sum }}{\left(x_{1}\left(t_{i},\vec{x}\left(0\right)\right)-V_{mem}\left(t_{i}\right)\right)}^{2}+x_{L+2}^{2}\left(t_{i}\right)\text{.}$$

The state of the neuron is represented by a vector $\vec{x}$with L+K+2 components. Vector components are $x_{1}$, the membrane voltage; $x_{2},\ldots ,x_{L}$, the gate variables of ion channels; $x_{L+1}$ and $x_{L+2}$, the Tikhonov regularization variable (Tikhonov, 1943; Abarbanel et al., 2010) and its time derivative; and $x_{L+2},\ldots ,x_{L+K+2}$, the model parameters. Model parameters are state variables whose time derivative is zero. Should a “parameter” depend on time as is sometimes the case in biology, this is easily accounted for by replacing zero with the rate of change of the parameter in Equation 3. The cost function is minimized subject to both equality constraints:


(4) $$\frac{dx_{l}}{dt}=F_{l}\;\left(x_{1},\ldots ,x_{L+K+2}\right),l=1,\ldots ,\left(L+K+2\right)\text{,}$$

specified by the neuron model, Equation 4, and the zero time derivatives of model parameters expressed as follows:


(5) $$F_{l}\left(\vec{x}\right)=\{\begin{array}{cc} -J/C-x_{L+1}\;\left(x_{1}-V_{mem}\right) & \;l=1\\ \frac{x_{l,\infty }-x_{l}}{\tau_{l}} & \;l=2,\ldots ,L\\ x_{L+2} & l=L+1\\ \mathrm{unspecified} & l=L+2\\ 0 & l=L+3,\ldots ,L+K+2 \end{array}$$

and inequality constraints (Equation 6):


(6) $$x_{l}^{\text{min}}\leq x_{l}\leq x_{l}^{\text{max}},l=1,\ldots ,\left(L+K+2\right)$$

bracketing the variation of membrane voltage, gate variables, regularization term and parameters (Equation 7),


(7) $$\left[x_{l}^{\text{min}},x_{l}^{\text{max}}\right]=\{\begin{array}{cccc} -100\mathrm{mV} & , & 50\mathrm{mV}\; & l=1\\ 0 & , & 1 & l=2,\ldots ,L\\ 0 & , & 1 & l=L+1\\ -1 & , & 1 & l=L+2\\ p_{l}^{\text{min}} & , & p_{l}^{\text{max}} & l=L+3,\ldots ,L+K+2 \end{array}$$

$J=J_{NaT}+J_{\mathrm{NaP}}+J_{K}+J_{A}+J_{Ca}+J_{BK}+J_{SK}+J_{HCN}+J_{\mathrm{Leak}}-J_{inj}$is the current per unit area of the neuron membrane. This includes 9 voltage-gated ionic currents, transient sodium (NaT), persistent sodium (NaP), delayed rectifier potassium (K), A-type potassium (A), calcium (Ca), large conductance calcium-activated potassium current (BK), small conductance calcium-activated potassium current (SK), hyperpolarization cation-activated cation current (HCN), leak current and the current injected to drive neuron oscillations, $J_{inj}$. We used the mathematical expressions of ionic currents given by Warman et al. (1994). The slow pump and exchange currents maintaining ionic gradients across the membrane are implicitly included in the constant reversal potentials, $E_{Na}$ and $E_{K}$, of Na^+^ and K^+^ ions. *C* is the membrane capacitance, $\tau_{l}$ the recovery time of ionic gate *l*, and $x_{l,\infty }$ is the steady-state value of gate variable $x_{l}$. This model of the hippocampal neuron has *K* = 67 parameters, *L* = 14 state variables. The user sets the parameter search range, $\left[p_{l}^{\text{min}},p_{l}^{\text{max}}\right]$ to the widest biologically plausible range for each parameter. Data assimilation outputs the optimal parameters and the state variables at *t* = 0 as $\vec{x}\left(0\right)$.

The equations of ionic currents are listed in Table 1.

**Table 1:** Equations of the conductance model.

Current	Gate dynamics	Gate activation
$J_{NaT}=g_{NaT}\;m_{\infty }\;h^{3}\;\left(E_{Na}-V\right)$,	$\frac{dh}{dt}=\frac{h_{\infty }\left(V\right)-h}{\tau_{h}\left(V\right)}$	$m_{\infty }\left(V\right)=0.5\left[1+\tanh\left(\frac{V-V_{m}}{\delta V_{m}}\right)\right]$ $h_{\infty }\left(V\right)=0.5\left[1+\tanh\left(\frac{V-V_{h}}{\delta V_{h}}\right)\right]$ $\tau_{h}\left(V\right)=t_{h}+\in_{h}\left[1-\tanh^{2}\left(\left(V-V_{h}\right)/\delta V_{\tau h}\right)\right]$
$J_{\mathrm{NaP}}=g_{\mathrm{NaP}}\;p_{\infty }\left(E_{Na}-V\right)$		$p_{\infty }\left(V\right)=0.5\left[1+\tanh\left(\frac{V-V_{p}}{\delta V_{p}}\right)\right]$
$J_{K}=g_{K}n^{4}\left(E_{K}-V\right)\text{,}$	$\frac{dn}{dt}=\frac{n_{\infty }\left(V\right)-n}{\tau_{n}\left(V\right)}$	$n_{\infty }\left(V\right)=0.5\left[1+\tanh\left(\frac{V-V_{n}}{\delta V_{n}}\right)\right]$ $\tau_{n}\left(V\right)=t_{n}+\in_{n}\left[1-\tanh^{2}\left(\left(V-V_{n}\right)/\delta V_{\tau n}\right)\right]$
$J_{A}=g_{A}\;a\;b\;\left(E_{K}-V\right)\text{,}$	$\frac{da}{dt}=\frac{a_{\infty }\left(V\right)-a}{\tau_{a}\left(V\right)}$ $\frac{db}{dt}=\frac{b_{\infty }\left(V\right)-b}{\tau_{b}\left(V\right)}$	$a_{\infty }\left(V\right)=0.5\left[1+\tanh\left(\frac{V-V_{a}}{\delta V_{a}}\right)\right]$ $\tau_{a}\left(V\right)=t_{a}+\in_{a}\left[1-\tanh^{2}\left(\left(V-V_{a}\right)/\delta V_{\tau a}\right)\right]$ $b_{\infty }\left(V\right)=0.5\left[1+\tanh\left(\frac{V-V_{b}}{\delta V_{b}}\right)\right]$ $\tau_{b}\left(V\right)=t_{b}+\in_{b}\left[1-\tanh^{2}\left(\left(V-V_{b}\right)/\delta V_{\tau b}\right)\right]$
$J_{Ca}=g_{Ca}\;s^{2}\;r\;\left(E_{Ca}-V\right)\text{,}$	$\frac{ds}{dt}=\frac{s_{\infty }\left(V\right)-s}{\tau_{s}\left(V\right)}$ $\frac{dr}{dt}=\frac{r_{\infty }\left(V\right)-r}{\tau_{r}\left(V\right)}$	$s_{\infty }\left(V\right)=0.5\left[1+\tanh\left(\frac{V-V_{s}}{\delta V_{s}}\right)\right]$ $\tau_{s}\left(V\right)=t_{s}+\in_{s}\left[1-\tanh^{2}\left(\left(V-V_{s}\right)/\delta V_{\tau s}\right)\right]$ $r_{\infty }\left(V\right)=0.5\left[1+\tanh\left(\frac{V-V_{r}}{\delta V_{r}}\right)\right]$ $\tau_{r}\left(V\right)=t_{r}+\in_{r}\left[1-\tanh^{2}\left(\left(V-V_{r}\right)/\delta V_{\tau r}\right)\right]$
$J_{BK}=g_{BK}\;c^{2}\;d\;\left(E_{K}-V\right)\text{,}$	$\frac{d\;c}{dt}=\frac{c_{\infty }\left(V,{\left[Ca\right]}_{\mathrm{in}}\right)-c}{\tau_{c}\left(V\right)}$	$c_{\infty }\left(V,{\left[Ca\right]}_{\mathrm{in}}\right)=0.5\left[1+\tanh\left\{\left\{V-V_{c}+130\left\{1+\tanh\left(\frac{{\left[Ca\right]}_{\mathrm{in}}}{0.2}\right)\right\}-250\right\}/\delta V_{c}\right\}\right]$ $\tau_{c}\left(V\right)=t_{c}+\in_{c}\left[1-\tanh^{2}\left(\left(V-V_{c}\right)/\delta V_{\tau c}\right)\right]$
	$\frac{d\;d}{dt}=\frac{d_{\infty }\left(V,{\left[Ca\right]}_{\mathrm{in}}\right)-d}{\tau_{d}\left(V\right)}$	$d_{\infty }\left(V,{\left[Ca\right]}_{\mathrm{in}}\right)=0.5\left[1+\tanh\left\{\left\{V-V_{d}+130\left\{1+\tanh\left(\frac{{\left[Ca\right]}_{\mathrm{in}}}{0.2}\right)\right\}-250\right\}/\delta V_{d}\right\}\right]$ $\tau_{d}\left(V\right)=t_{d}+\in_{d}\left[1-\tanh^{2}\left(\left(V-V_{d}\right)/\delta V_{\tau d}\right)\right]$
$J_{SK}=g_{SK}\;w_{\infty }\;\left(E_{K}-V\right)$,		$w_{\infty }\left({\left[Ca\right]}_{\mathrm{in}}\right)=0.5\left[1+\tanh\left\{\left\{V-V_{w}+130\left\{1+\tanh\left(\frac{{\left[Ca\right]}_{\mathrm{in}}}{0.2}\right)\right\}-250\right\}/\delta V_{w}\right\}\right]$
$J_{HCN}=g_{H}\;z\;\left(E_{HCN}-V\right)$	$\frac{dz}{dt}=\frac{z_{\infty }\left(V\right)-z}{\tau_{z}\left(V\right)}$	$z_{\infty }\left(V\right)=0.5\left[1+\tanh\left(\frac{V-V_{z}}{\delta V_{z}}\right)\right]$ $\tau_{z}\left(V\right)=t_{z}+\in_{z}\left[1-\tanh^{2}\left(\left(V-V_{z}\right)/\delta V_{\tau z}\right)\right]$
$J_{\mathrm{Leak}}=g_{L}\left(E_{L}-V\right)$.		
Calcium dynamics	$\frac{d{\left[Ca\right]}_{\mathrm{in}}}{dt}=\frac{{\left[Ca\right]}_{\infty }-{\left[Ca\right]}_{\mathrm{in}}}{\tau_{ca}}-\frac{J_{Ca}}{2tF}$	

Among the *K* = 67 parameters are the ion channels conductances ($g_{\alpha }$), activation thresholds ($V_{\beta }$), activation slopes ($\delta V_{\beta }$), and recovery time parameters ($t_{\beta },\in_{\beta },\delta V_{\tau \beta }$) where α≡{NaT,NaP,K,A,Ca,BK,SK, HCN, Leak} and $\beta \text{≡}\left\{m,h,p,n,a,b,s,r,c,d,w,z,{\left[Ca\right]}_{\mathrm{in}}\right\}$. The Na reversal potential was set to $E_{Na}$ = +42 mV and HCN reversal potential to $E_{HCN}=-43$mV while the other reversal potentials $E_{K},E_{Ca},E_{L}$ were estimated by RPDA. The BK and SK currents are calcium activated potassium currents (Warman et al., 1994). The dynamics of the internal calcium concentration (last equation) depends on the equilibrium concentration ${\left[Ca\right]}_{\infty }$, the Ca recovery time constant ($\tau_{Ca}\approx 1.5$ ms), Faraday’s constant (F), and the thickness of the membrane across which $Ca^{2+}$ fluxes are calculated (t≈1μm). The membrane capacitance was set to $C=1\;\mu F.{\mathrm{cm}}^{-2}$. The effective area of the soma through which the current was injected was also estimated.

The RPDA algorithm, re-injects membrane voltage data in the optimization problem at the beginning of every block of *M* data-points. This means that the membrane voltage state variable $x_{1}$ is replaced with $V_{mem}$ at time points *t*_0_, *t*_M-1_, *t*_2M-1_ in the linearized expression of the equality constraints:


$$x_{l}\left(t_{i+2}\right)=x_{l}\left(t_{i}\right)+\Delta t\;\left[\frac{1}{3}F_{l}\left(\vec{x}\left(t_{i}\right)\right)+\frac{4}{3}F_{l}\left(\vec{x}\left(t_{i+1}\right)\right)+\frac{1}{3}F_{l}\left(\vec{x}\left(t_{i+2}\right)\right)\right]\text{,}$$

(8) $$x_{l}\left(t_{i+1}\right)=\frac{1}{2}\left[x_{l}\left(t_{i}\right)+x_{l}\left(t_{i+2}\right)\right]+\frac{\Delta t}{4}\left[F_{l}\left(\vec{x}\left(t_{i}\right)\right)-F_{l}\left(\vec{x}\left(t_{i+2}\right)\right)\right]\text{,}$$

$$for\;l=1,\ldots ,\left(L+K+2\right)\;\mathrm{and}\;i=0,\ldots ,\left(N-2\right)\text{.}$$

where Δt=T/N is the time interval between consecutive data points in the assimilation window (0.01 ms). At other times the state vector is propagated normally from $t_{i}$ to $t_{i+2}$ by Equations 8. The substitution of $V_{mem}$ also impacts the first and second derivatives of the objective function with respect to $x_{1}$. Where the cost function ceases to depend on $x_{1}$, its derivatives with respect to $x_{1}$ vanish. We explicitly set these derivatives to zero at times $t_{0},t_{M-1},t_{2M-1}\ldots$. This produces a discontinuity in $x_{1}\left(t\right)$at the beginning of each *M*-block which is the trade-off for imposing the bias to the solution. Our piecewise fit of data has similarities with the multiple shooting approach proposed by Bergmann et al. and Zimmer and Sahle (Bergmann et al., 2016; Zimmer and Sahle, 2015). However our RPDA method performs piecewise assimilation of blocks of data through redefined constraints, whereas the Zimmer and Sahle’s approach redefines the cost function.

## 4 Results

### 4.1 Selective switching of ion channels

We have performed current clamp measurements in single hippocampal cells under the protocol depicted in Figure 2. Whole cells measurements were performed in brain slices of Han Wistar rats. The cell was pharmacologically isolated from the activity of neighboring cells through the application of (μM) kynurenate 3, picrotoxin 0.05 and strychnine 0.01 to inhibit glutamatergic, GABAergic and glycinergic neurotransmission, respectively. A series of 5 s long epochs were first recorded under varying current protocols (Morris et al., 2024). A pharmacological inhibitor was then applied to block a specific ion channel. The same series of current protocols was then applied post drug and the neuron oscillations were recorded (Figure 2). We will report here on two ion channel antagonists, iberiotoxin (IbTX) and 4-aminopyridin (4-AP), that selectively inhibit the BK and A-type current.

We then used RPDA to generate one set of 67 model parameters before drug and after drug. In each case, the same 800 ms long section of the epoch was assimilated. In order to account for the statistical fluctuations in the parameter field induced by data and model error, we generated multiple sets of parameters (*R* = 15 for IbTX; *R* = 19 for 4-AP) from *R* assimilation windows offset in time (Figure 2). The dispersion of parameters and subsequently the dispersion of ionic current waveforms reconstructed from then is useful to evaluate the prediction error on the changes predicted by RPDA.

### 4.2 Predicting membrane voltage oscillation before and after applying an inhibitor

**Figure 3:**
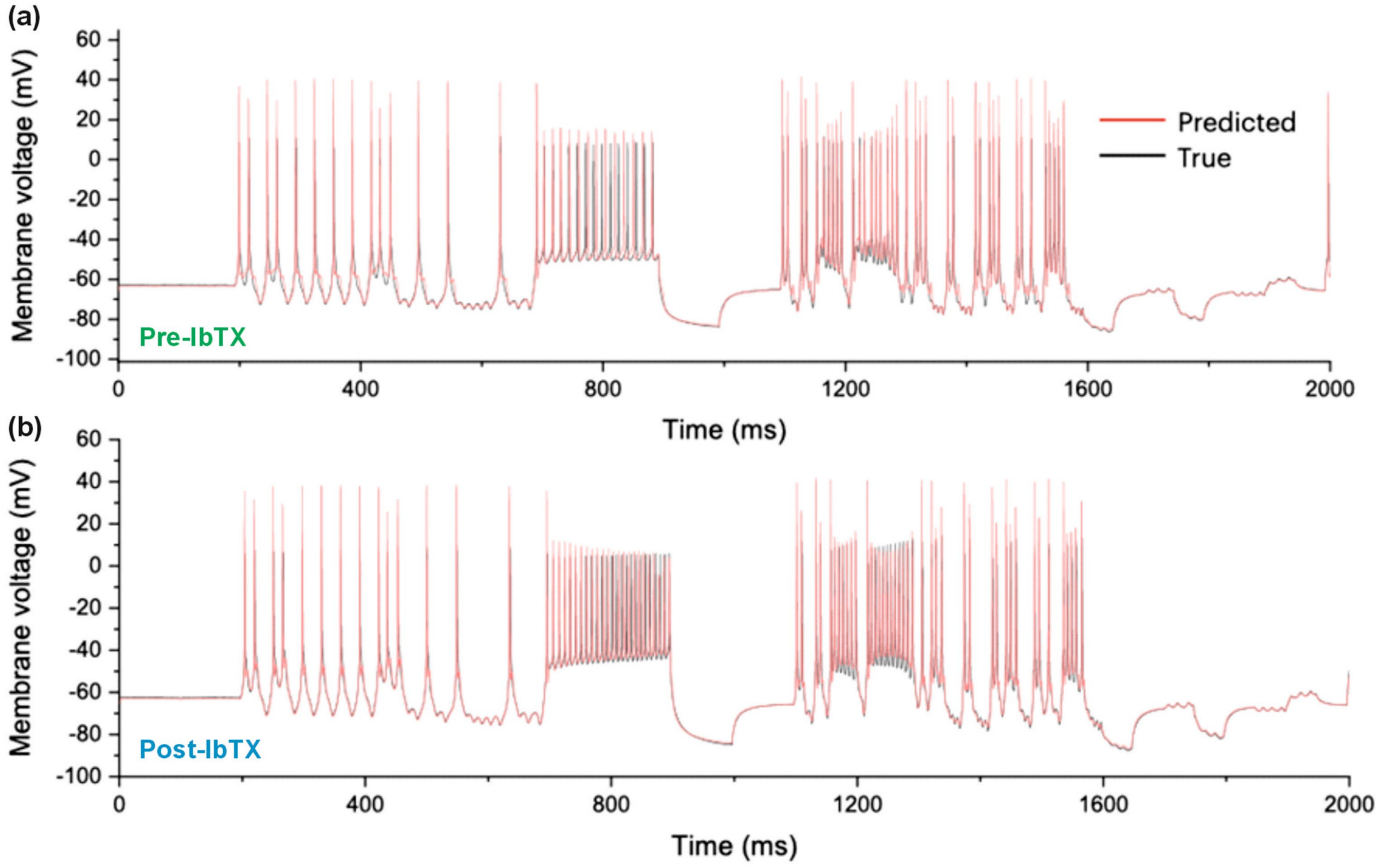
Measured and Predicted membrane voltage. **(A)** 2,000 ms long epoch comparing the experimental membrane voltage oscillations of a CA1 hippocampal neuron (black line) to the membrane voltage oscillations predicted by the conductance model (red line). The first 1,000 ms of this epoch are also shown in Figure 1 with the stimulation current. **(B)** Measured and predicted membrane voltage after the application of iberiotoxin (IbTX: 100 nM).

We performed a first validation test of the RPDA method by forward-integrating the current protocol of Figure 2A with two models completed with pre-drug and post-drug parameters, and predicting the membrane voltage oscillations of the neuron (Figure 3, red lines). The predicted traces show a very good agreement with the experimental membrane voltage oscillations (Figure 3, black lines). Some discrepancy is observed in the region of dense spikes at about 800 ms where the current excitation is constant. In this region, the precise timing of neuron spikes is less reproducible because of the stochastic fluctuations of the neuron. This discrepancy does not imply the model is wrong. Note that in other regions at about 400 ms and 1,400 ms where the current varies faster than stochastic fluctuations, the timing of spikes is very reproducible and the agreement with the model is excellent. Mainen and Sejnowski (1995) have previously pointed out the lack of reproducibility of a neuron to tonic stimulation and this is what we are seeing here. Nogaret et al. (2016), (Fig. S7 [blue arrows]) have further shown that models constructed by data assimilation correctly predict spikes that are logically expected to occur for a given stimulation pattern but may be missing because of internal stochastic fluctuations. The consistent error in model predictions is the overestimation of spike amplitudes.

### 4.3 Predicting the selectivity and potency of known inhibitors

**Figure 4:**
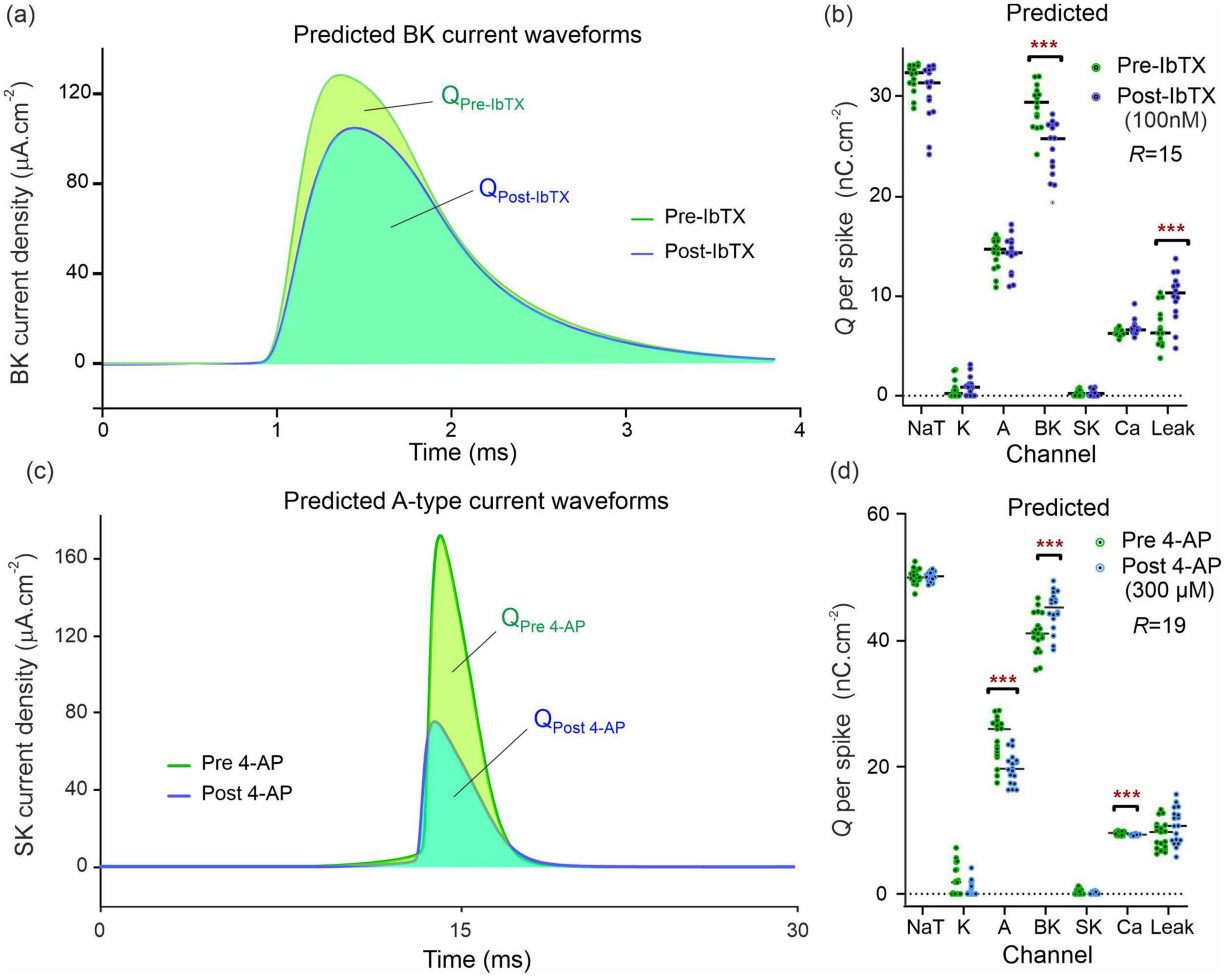
Predicted ion current waveforms pre- and post-inhibition. **(A)** Predicted BK current waveforms before and after application of Iberiotoxin (100 nM). The pre-IbTX current trace is the average of 15 waveforms obtained by integrating the conductance model with parameters {$p_{1,\ldots ,15}^{Pre}$}. The post IbTX traces were similarly obtained from parameters {$p_{1,\ldots ,15}^{\mathrm{Post}}$}. **(B)** Predicted ionic charge transferred through each ion channel of the CA1 neuron before and after IbTX. Each dot is obtained from the integration of a BK current waveform predicted by the model constructed from one of the R = 15 assimilation windows in Figure 2. The green dots are the charge predictions pre-drug and the blue dots are the charge predictions after 100 nM IbTX was applied. The median charge is shown by the horizontal bars. Asterisks (***) indicate multiplicity adjusted q values from multiple Mann–Whitney U tests using a False Discovery Rate approach of 1%. **(C)** Predicted A-type current waveforms before and after application of the 4-aminopyridin. **(D)** Predicted ionic charge transferred through each ion channel of the CA1 neuron before and after 4-AP. The data in **(A,B)** were generated from one animal and the data in **(C,D)** from another animal. These data are exemplar of the recordings taken on the 13 animals we have studied in total.

Once the 2*R* sets of 67 parameters (pre- and post-drug) were obtained, these were inserted in the neuron equations, Equation 5 (Warman et al., 1994). The completed models were then forward integrated to obtain the ionic current waveforms of all 9 ion channels. The voltage traces predicted by each completed model were compared to the experimental traces in the study by Morris et al. (2024). The good agreement achieved pre- and post-drug provides an intermediate validation point for the optimized neuron models. Although this agreement is necessary, it is not sufficient for claiming to infer information on ion channels: Wrong models can also predict the membrane voltage. Figure 4A shows the BK current waveforms pre- and post-IbTX predicted at the site of one action potential. The net charge transferred through each ion channel per action potential was obtained from the areas under the ionic current waveforms and plotted in Figure 4B. The analysis of neurons subjected to iberiotoxin (IbTX; 100 nM; Figure 4B; *R* = 15 pre-drug and post-drug) predicted a *12.1% reduction* in median and 14.8% reduction in mean BK-mediated charge per action potential. This was one statistical discovery across all channels in the drug-applied data (Figure 4B; U = 25; q < 0.01; mean ranks 21.2 [pre-IbTX], 9.8 [post-IbTX]). Charge transfer decreased from 29.4 nC · cm^−2^ to 25.9 nC · cm^−2^. A compensatory increase in leak current was also identified, likely due to decreased K+ permeability caused by IbTX (U = 37.5; *q* < 0.01; mean rank 10.5 [pre IbTX], 20.5 [post-IbTX]). Leak charge transfer increased from 6.3 nC · cm^−2^ to 10.3 nC · cm^−2^, with a mean increase of 46%. There were no statistical discoveries for any other channels. This demonstrates that models constructed by RPDA correctly predict the selectivity of IbTX. Figure 4B predicts the reduction in charge transfer through the BK channel targeted by IbTX.

Similarly, we reconstructed the 9 ionic current waveforms through each of the 9 ion channels before and after applying 4-AP. The waveform of the A-channel targeted by 4-AP is shown in Figure 4C. The amount of ionic charge transferred pre- and post-drug for each ion channel was then obtained by integrating the current waveform through each ion channel. The results are plotted in Figure 4D. Following the application of 4-Aminopyridine (300 μM; Figure 4D; *R* = 19 pre-drug; *R* = 18 post-drug), our completed models predicted a reduction in charge transfer mediated by A-type K+ channels (Figure 4D; U = 52; q < 0.001; mean rank 25.3 [pre 4-AP], 12.4 [post 4-AP]). Median charge transfer dropped from 26.1 nC · cm^−2^ to 19.7 nC · cm^−2^ with a 19.0% mean reduction. In addition, the model predicts a 10.0% increase in median charge transfer (8.8% mean) through the BK-channel (U = 73; q < 0.01; median charge 41.2 nC · cm^−2^ [pre 4-AP], 45.3 nC · cm^−2^ [post 4-AP]); and a reduction in Ca^2+^-mediated charge transfer (U = 79; q < 0.01; mean rank 23.8 [pre 4-AP], 13.9 [post 4-AP]). Ca^2+^-mediated charge dropped from 9.65 nC · cm^−2^ to 9.23 nC · cm^−2^ with a mean reduction of 3.0% mean. Figure 4D predicts the reduction in charge transfer through the A-type K^+^ channels targeted by 4-AP. RPDA predictions uniquely quantify the collateral effects of 4-AP on BK and Ca channels because this is a single shot estimation of alterations across all ion channels in the cell. These collateral alterations are consistent with modifications of the electrochemical driving force by the antagonist which alters current flow through the other ion channels, in particular the calcium mediated potassium channel (BK).

We finally compared the mean/median degree of ion channel block predicted by RPDA with the nominal degree of block expected for each inhibitor at the concentration applied. The results are summarized in Table 2. A first observation is that both BK and A-type ion channels may express different sub-types of ion channels. These exhibit different sensitivities to antagonists. With this caveat, RPDA predictions are broadly consistent with the $\alpha +\beta_{1}$ subunit of the BK channel being predominantly blocked. Similarly, the RPDA-predicted block is consistent with the Kv1.4 subunit of the A-type current. In addition, the coefficient of variation of the statistical distribution of predicted charge transfer (Figures 3C,D) suggests that RPDA is accurate within ±11%.

**Table 2:** Inhibition of ionic currents predicted versus nominal.

Drug	Concentration applied	Channel type (rodent)	Expected degree of block at applied concentration	Median / mean predicted inhibition (results)	Relative St. dev of mean inhibition (Results)
Iberio-toxin	100 nM	BK [α subunit only]	86%	12.1% / 14.8%	21.3%
		BK [α + β1]	12%		
		BK [*α* + β3]	77%		
		BK [α + β4]	Insensitive		
4-AP	300 μM	A-type [Kv1.4] (+)	25% [selectively expressed in xenopus oocytes]	24.3% / 19.0%	21.1%
		A-type [Kv4.2] (++)	13% [selectively expressed in xenopus oocytes]		

## 5 Discussion

The above data demonstrate the effectiveness and accuracy of RPDA as a single shot method to quantify ion channel alterations across the complement of ion channels of a cell. RPDA estimates model parameters with nearly perfect accuracy and reliability *when the model is known*. The main limitation to prediction accuracy when modelling real data is the lack of knowledge of the true biological equations. Model error introduces correlations among some parameters with tend to cancel out in the calculation of ionic currents. As a result, the current waveforms reconstructed from these parameters are more reliable predictors than the underlying parameters. Data error also introduces uncertainty in the parameter field. For all practical purposes this error is often small as modern current clamp amplifiers produce very high-quality neuron recordings. In other cases, data error can be traced to a well identified source such as noise, space clamp, or liquid junction potential and corrected.

The cost function in Equation 3 is a reduction of the general cost function (Abarbanel, 2022) that includes both data error and model error. This is expressed as follows:


(9) $$\begin{array}{l} c\left(\vec{x}\left(0\right)\right)=\frac{1}{2}\overset{N}{\underset{i=0}{\sum }}Rd_{i}^{-1}{\left(x_{1}\left(t_{i}\right)-V_{mem}\left(t_{i}\right)\right)}^{2}\\ \;+\frac{1}{2}\overset{L+K+2}{\underset{l=1}{\sum }}\overset{N}{\underset{i=1}{\sum }}Rm_{l\times i}^{-1}{\left(x_{l}\left(t_{i}\right)-G_{l}(\vec{x}\left(t_{i}\right),\vec{x}\left(t_{i-1}\right)\right)}^{2} \end{array}$$

The first term (data error) in Equation 9 is weighted by the covariance matrix Rd which describes the degree of confidence on each measurement. This matrix is diagonal since consecutive current clamp measurements are uncorrelated. In addition, all diagonal terms are the same since each membrane voltage measurement carries the same uncertainty. The first term then reduces to our first term in Equation 3. When the model is known, the second term vanishes, and the cost function reduces to the least square sum of Equation 3.

The second term incorporates model error as $G_{l}\left(\vec{x}\left(t_{i}\right),\;\vec{x}\left(t_{i-1}\right)\right)\approx x_{l}\left(t_{i-1}\right)+\Delta tF_{l}^{\prime }\left(\vec{x}\left(t_{i}\right)\right)$where $F_{l}^{\prime }\left(\right)$ are the empirical conductance equations approximating the unknown true equations $F_{l}\left(\right)$. A current challenge in evaluating this term is the lack of method for calculating the model error covariance matrix $R_{i\times i}$. This remains an open problem and is the reason for not including this term in Equation 3.

An advantage of RPDA is that prior knowledge of the expressed ion channels can help but is not mandatory. Over-specifying the model by including unexpressed ion channels is of no consequence as RPDA assigns zero conductance to the absent ion channels. Remarkably recent simulations (Wells et al., 2024) show that even when the model contains mild error, RPDA still assigns residual conductance to the unexpressed ion channel. In particular, the unexpressed currents do not compensate for model error in the expressed ion channels by being assigned a finite conductance. More work is needed to investigate the range of model error within which RPDA successfully deconvolutes ionic currents. However, the present experimental data show that RPDA is sufficiently accurate to predict the selectivity and potency of known pharmacological inhibitors. Further improvements to the accuracy of RPDA will require the incorporation of a model error correction algorithm. This would reduce the uncertainty on estimated parameters – not just ionic currents—and allow building larger models such as central pattern generators, or multicompartmental models to account for neuronal morphology (Nandi et al., 2022).

RPDA presents a number of advantages compared to genetic, evolutionary or Metropolis-Hastings type algorithms. As a gradient descent method, RPDA requires fewer repeated evaluations of the cost function as the size of the problem increases. Fitting biological neurons requires large multichannel models with a high-dimensional parameter space, *K* > 50. The cost of these algorithms in terms of computer time becomes prohibitive for realistic conductance models. In RPDA, the Jacobian and Hessian of constraints and cost function are known. They are computed in analytical form after symbolic differentiation. Therefore convergence in high-dimensional parameter space is comparatively fast. The stopping criterion is also clear cut in RPDA. The evaluation of the cost function at the global minimum is typically 5 orders of magnitude smaller than at local minima (Taylor et al., 2020). This marked differentiation between global and local minima is one reason why RPDA achieves ∼100% convergence in *well-posed* problems. The local minima of *ill-posed* problems are specifically handled by the piecewise reinjection of data in RPDA. In contrast genetic algorithms have a tendency to converge to local minima.

The morphology of neuronal trees could be modelled by using multi-compartmental models in RPDA, for instance to infer information on calcium channel activity in dendrites. Introducing additional neuron compartments in the model is tractable within RPDA in the same way as adding supplementary ion channels. Transmission line delays associated with dendrites and axons is known to alter the shape of action potentials. This said, single compartment models have excelled in predicting membrane voltage dynamics (Brookings et al., 2014; Nogaret et al., 2016). It may be argued that the aggregate duration of all action potentials across the assimilation window is small compared to the time spent in the subthreshold state. The shape of action potential is thus less important than spike width, height, inter-spike intervals and sub-threshold oscillations in determining model parameters. The presence of dendrites and axons is likely to be accounted for by an *effective* inactivation time constants which integrates the real inactivation time constants and the delays introduced by attachments to the soma. This brings us to the important conclusion of our work (Wells et al., 2024) that at the present time ionic currents reconstructed from estimated parameters carry a higher degree of confidence than the parameters.

## Data Availability

The original contributions presented in the study are included in the article/Supplementary material, further inquiries can be directed to the corresponding author.
