# Supplementary material for: Estimation of ionic currents and compensation mechanisms from recursive piecewise assimilation of electrophysiological data
Source: Front Comput Neurosci. 2025 Mar 4;19:1458878. doi: 10.3389/fncom.2025.1458878 (PMC11913807; doi:10.3389/fncom.2025.1458878)
Supplement: Supplementary file 1 [file Data_Sheet_1.zip › README.docx]

Recursive piecewise data assimilation first requires Installing IPOPT from:
<https://github.com/coin-or/Ipopt>
including the MA97 linear solver from:
<https://www.hsl.rl.ac.uk/catalogue/hsl_ma97.html>

Instructions for use on the LINUX operating system:

- Make three directories **mkdir** : /code, /data, and /output
- In /code, upload
- bounds.txt
  contains (lower bound, initial guess, upper bound) of the search range of state variables, control variable, derivative of control variable, and model parameters in the same order as specified in the dys_sys_RVLM.text file.
- compileNaM2.py
  Python file doing symbolic differentiation of the Jacobian and Hessian of the cost function and the conductance model and generating the C++ file that will be compiled with “Make”.
- dyn_sys_RVLM.txt
  this file specifies the 7 state variables, parameters of the conductance model, and the system of equations of the conductance models. The first line is the right-hand side of the dV/dt equation in the Hodgkin-Huxley model. The following 6 equations are the right-hand side of the gate equations (i.e. dm/dt, dh/dt etc.)
- ipopt.opt
  Output log
- methods_mss.py
- called by the compile.py file
- mss_info.txt
- recursion parameters in RPDA.
- outputfiles_mss.py
- called by the compile.py file
- problem_info.txt
  specifies the number of data points in the window, number of points to skip at the beginning, whether to use an adaptive step size or equal intervals across the assimilation window, base step size, the file describing the distribution of intervals when the adaptive option is selected.
- In /data, upload
- Iapp.txt
  Data file containing the current protocol time series.
- V.txt

Data file containing the membrane voltage oscillations to fit.

- Change the points observed (100001) and skip (0) in problem_info.txt
- Run **python compileNaM2.py**
- This creates a new set of files with .cpp and .hpp extensions
- Also, a Makefile
- **make**
- This will take all the files and read it
- By creating an executable file, errm1skip01c.exe in this case
- Run the code!
- **nohup ./errm1skip01c.exe &**
- nohup – means no hang up which means keep running even when I sign out
- & - to run the code in the background
- **tail -f nohup.out**
- To check if the code is still running and also check the objective values.
- Objective values should be low, as it tells the data fit
- If the objective is too high, you may have made some errors and run the code again
